# Chitosan functionalized nitrogen-doped carbon dot nanocomposites: a turn-on sensor for fluoride detection

**DOI:** 10.1039/d5ra04745e

**Published:** 2025-09-17

**Authors:** Sujal, Raj Singh, Suman Swami, Shikha Bhogal

**Affiliations:** a Department of Chemistry, University Institute of Sciences, Chandigarh University Mohali-140413 Punjab India sumanswami1994@gmail.com; b Virginia Tech-TIET Center of Excellence in Emerging Materials, Thapar Institute of Engineering & Technology Patiala – 147004 India shikha.bhogal@thapar.edu

## Abstract

The fluoride anion plays a vital role in human health by preventing dental caries and enhancing bone strength. However, excessive intake beyond the permissible limit poses a serious health risk. This underscores the critical need for accurate and routine monitoring of fluoride levels in water supplies. Traditional methods of fluoride detection are associated with many limitations and high hydration enthalpy of fluoride (Δ*H* = −504 kJ mol^−1^), which additionally makes it challenging to design effective aqueous-phase fluorescent probes for fluoride. In this study, we report chitosan-functionalized N-doped carbon dots (N-CDs@C) as an environmentally friendly, cost-effective, and fluorescent probe for the selective and sensitive detection of fluoride ions in aqueous media. The interaction between N-CDs@C and fluoride likely occurs *via* hydrogen bonding and electrostatic interaction, which results in a fluorescence “turn-on” response for fluoride. The N-CDs@C probe exhibited a strong emission enhancement at 455 nm upon fluoride binding, with excellent selectivity, anti-interference performance, and a detection limit as low as 0.01 μM within a linear range of 0.12–0.50 μM. The practical utility of this sensor was demonstrated through successful application in real samples, including toothpaste and tap water with excellent recoveries ranging from 98.67–99.27% (RSD <2%).

## Abstract 2

Chitosan-functionalized nitrogen-doped carbon dots (N-CDs@C) were synthesised as an environmentally-friendly fluorescence probe. N-CDs@C was employed as “turn-on” sensor for the detection of fluoride anion.

## Graphical abstract

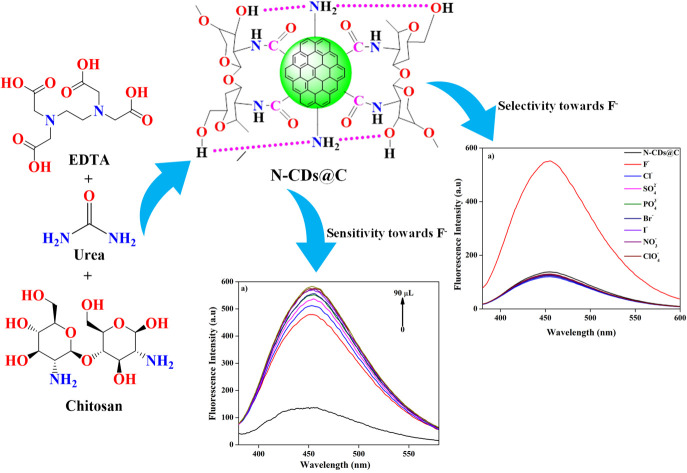

## 1. Introduction

Fluoride (F^−^) is a crucial anion that significantly contributes to human health.^1^ Fluoride, in appropriate amounts, is recognised for its role in preventing dental caries and enhancing bone strength, establishing it as an essential component in toothpaste along with particular medications for osteoporosis.^2–4^ In addition to its biological significance, fluoride functions as an effective catalyst in both inorganic and organic synthesis and is utilized in the detection of chemical warfare agents (soman and sarin), and is widely utilized in uranium refinement.^5,6^ Fluoride is an essential anion for human health, but, beyond the permissible limit (1.5 mg L^−1^), intake of fluoride may pose serious health risks, including gastric and kidney disorders, urolithiasis, skeletal and dental fluorosis.^7–9^ More seriously, a high level of fluoride ions can disturb the synthesis of protein and DNA, impair the immune system, and even lead to fatal outcomes.^10^ Due to the fact that drinking water is the primary source of fluoride intake in the human body, a high incidence of dental caries (70–90%) is associated with fluoride levels below 0.5 ppm. Conversely, levels exceeding 1 ppm can result in dental mottling, and concentrations above 4 ppm significantly increase the risk of fluorosis.^7,8^ Consequently, considering the contradictory behaviours of fluoride at different concentrations, the World Health Organisation (WHO) advises that fluoride levels in drinking water should not surpass 1.5 mg L^−1^,^11^ while the US Environmental Protection Agency sets a maximum concentration restriction of 4.0 mg L^−1^.^12^ These guidelines highlight the critical need for accurate and routine monitoring of fluoride levels in water supplies.

Conventional fluoride detection methods, including ion chromatography,^13,14^ ion selective electrodes,^15,16^ electro-chemical assays,^17,18^ and colorimetry^19–21^ are often associated with some limitations such as complex operating procedures, expensive instrumentation, and expert handling required. Fluorescence-based sensing has emerged as a promising alternative due to its high sensitivity and rapid response. However, the high hydration enthalpy of fluoride (Δ*H* = −504 kJ mol^−1^),^22,23^ makes it challenging to design effective aqueous-phase fluorescent probes for fluoride.^24^ Moreover, many reported fluoride sensors are small organic molecules^10^ (anthracene, benzothiazole, BODIPY, coumarin, hydrazone, imidazole, and naphthalimide). For example, Xiaoliang Dong *et al.* have reported anthracene-derived 1-(anthracen-9-ylmethyl)urea and 1-(anthracen-9-ylmethyl)thiourea as a turn-on fluorescent probe for fluoride sensing.^25^ Serkan Erdemir and Ozcan Kocyigit developed benzothiazole-bisphenol A as a Schiff base sensor for fluoride sensing in CH_3_CN.^26^ Juan Liu *et al.* utilized a BODIPY derivative as a fluorometric and colorimetric sensor for fluoride sensing in CHCl_3_ medium,^27^ and Li Yun Zhao *et al.* reported a 1,8-Naphthalimide-derived colorimetric sensor (yellow–red) for fluoride in DMSO.^28^ All these studies require elaborate synthesis and organic solvents, raising concerns about environmental impact and limited water compatibility.^29,30^ As a result, there is a significant need to develop a fluorescent sensor that is environmentally friendly, easy to use, highly sensitive, and capable of accurately detecting fluoride in aqueous phases.

Recently, a new class of nanomaterial, namely carbon dots (CDs), has garnered significant attention for optical sensing due to their remarkable fluorescent properties, strong photostability, excellent biocompatibility, and ease of synthesis.^31,32^ Surface modification of CDs through heteroatoms (N, S, and P) doping or biopolymers functionalization further enhanced their photoluminescence properties, chemical stability, and selective interaction sites.^33–35^ Chitosan, a naturally abundant biodegradable biopolymer derived from chitin, offers additional benefits such as muco-adhesiveness and abundant amino and hydroxyl functional groups, which can serve as binding sites for anions.^36^

In the present study, we report the chitosan-functionalized N-doped carbon dots (N-CDs@C) as an environmentally friendly, cost-effective, and fluorescent probe for the selective and sensitive detection of fluoride ions in aqueous media. The interaction between N-CDs@C and fluoride likely occurs *via* hydrogen bonding and electrostatic interaction, which results in a fluorescence *“turn-on”* response for fluoride. The N-CDs@C probe exhibited a strong emission enhancement at 455 nm upon fluoride binding, with excellent selectivity, anti-interference performance, and a detection limit as low as 0.01 μM within a linear range of 0.12–0.50 μM. The practical utility of this sensor was demonstrated through successful application in real samples, including toothpaste and tap water.

## 2. Experimental sections

### 2.1. Chemicals and instrumentation

All details of chemicals and instrumentation have been provided in Tables S1 and S2 of (SI).

### 2.2. Synthesis of chitosan functionalized nitrogen-doped carbon dots (N-CDs@C)

The synthesis of N-CDs@C involves two major steps: (i) synthesis of N-CDs and (ii) functionalization of CDs with chitosan. Firstly, N-CDs were synthesized by a solid pyrolytic method containing urea (9 g) and ethylene diamine tetraacetic acid (EDTA) (1 g). The mixture was then heated in a crucible at 200 °C for 1 h in an oven. The final product, as black precipitate, was then dispersed in distilled water (DW) and filtered using Whatman filter paper, and then dried and stored for further analysis. For the functionalization with chitosan, chitosan (200 mg) and acetic acid (5 mL) were added to a 100 mL DW and sonicated for 15 min. The solution was then subjected to magnetic stirring for 1 h, and then N-CDs (100 mg) were added, and the reaction was allowed to stir for 5 h. After the completion of the reaction, the synthesized N-CDs@C was centrifuged and then dried at 50 °C.

### 2.3. Detection of fluoride ion

For the detection of F^−^ ion, firstly, 10 mg of N-CDs@C was dispersed in 5 mL of methanol to prepare a stock solution. Then, in a glass vial, 200 μL of N-CDs@C, 10 μL of F^−^ (3.8 × 10^−5^ M), 100 μL of pH 8 solution, and diluted with DW to make the final volume of 3 mL. After 5 min of incubation, the solution was analyzed at an emission wavelength of 455 nm (*λ*_ex_ = 370 nm) with a slit width of 10 nm. The selectivity of N-CDs@C was also tested towards different anions, including fluoride (F^−^), chloride (Cl^−^), sulfate (SO_4_^2−^), phosphate (PO_4_^2−^), bromide (Br^−^), iodide (I^−^), nitrate (NO_3_^−^), and perchlorate (ClO_4_^−^). For this, 10 μL of different anions (0.5 μM) were mixed with N-CDs@C (200 μL), and after 5 minutes, the fluorescence emission spectra were checked with an excitation wavelength of 370 nm and emission wavelength of 455 nm.

### 2.4. Real sample analysis

Two different samples have been taken for the real-time analysis of F- ion by N-CDs@C, including toothpaste and tap water. Tap water was collected from our laboratory, Chandigarh University, Gharaun (India). Toothpaste has been purchased from a local shop in Kharar, Punjab, India. For the toothpaste sample preparation, 5 g of toothpaste was immersed in 10 mL of DW and sonicated for 30 min. The extracted supernatant was further diluted with DW. All the samples were filtered through Whatman grade 1 filter paper before analysis.

## 3. Results and discussion

### 3.1. Synthesis of N-CDs@C

**Fig. 1:**
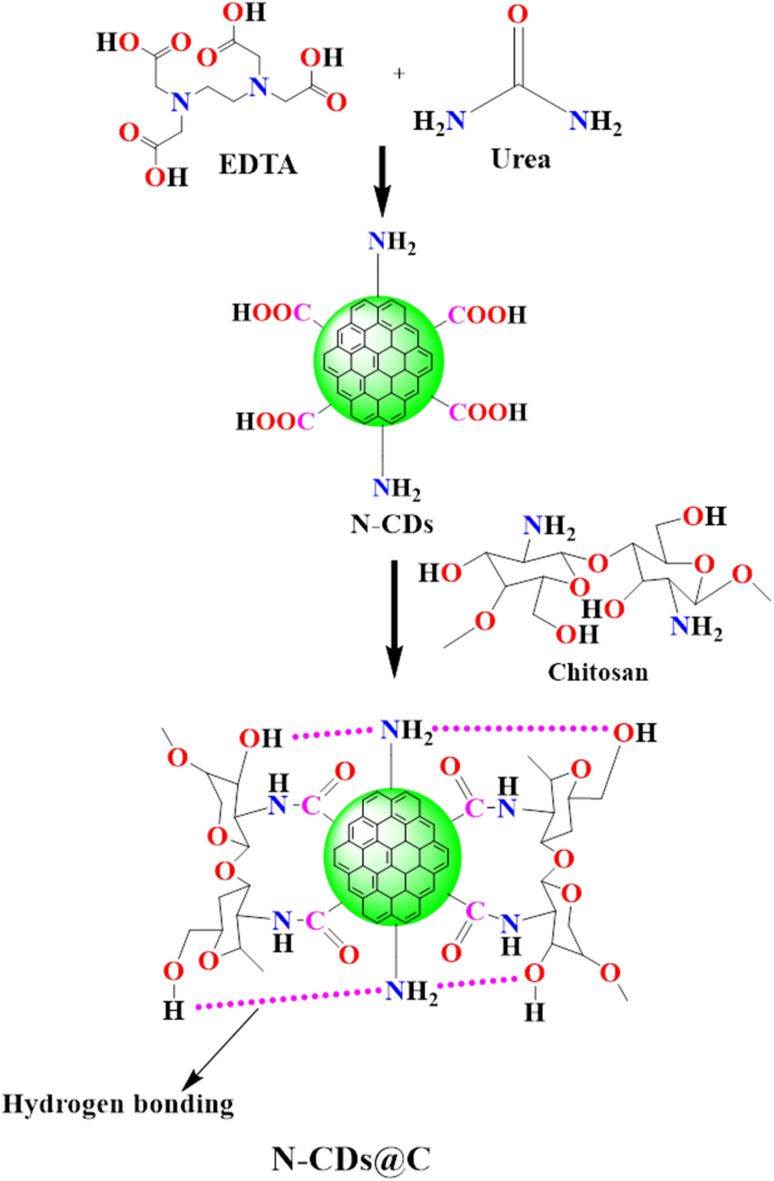
Schematic illustration of the synthesis of chitosan functionalized carbon dots (N-CDs@C).

The N-CDs@C composite was developed through two principal stages: the hydrothermal synthesis of N-CDs and their subsequent functionalization with chitosan. The synthesis process involved hydrothermal carbonization, dehydration, and polymerization of urea and EDTA, resulting in nitrogen-doped carbon dots (N-CDs) that possess various functional groups, including –COOH, –NH_2_, and –OH. The synthesized N-CDs were covalently linked to chitosan *via* the formation of amide bonds between the –COOH group of N-CDs and the –NH_2_ group of chitosan. Additionally, hydrogen bonding and electrostatic interactions further facilitated the association between N-CDs and chitosan (Fig. 1). The resulting N-CDs@C composite demonstrated improved photoluminescence properties, chemical stability, and selective interaction sites, rendering it particularly advantageous for sensing applications, especially for fluoride ions.

### 3.2. Characterization

**Fig. 2:**
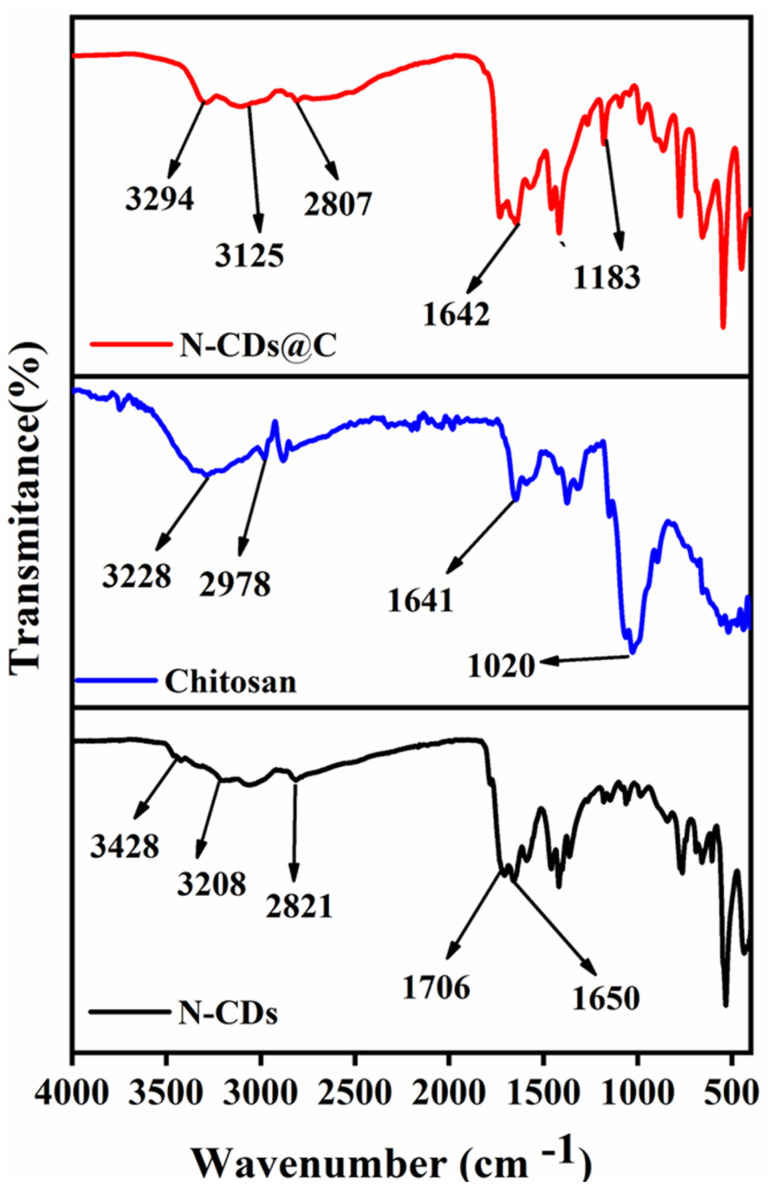
FTIR spectra of the synthesized materials: N-CDs, chitosan, and N-CDs@C.

The functional group analysis of N-CDs, chitosan, and N-CDs@C was confirmed by FT-IR. For N-CDs, the large absorption bands at 3428, 3208, 2871, 1706, and 1650 cm^−1^ correspond to the O–H stretch, N–H stretch, C–H stretch, C=O stretch, and C=C stretch, respectively (Fig. 2). On the other hand, for chitosan, the main peaks appeared at 3228, 2978, 1641, and 1020 attributed to O–H and N–H stretch, C–H stretch, C=O stretch, C=C stretch, and C–O vibrations, respectively (Fig. 2). Upon the formation of N-CDs@C, some spectral changes have been observed (Fig. 2). The broad band shifted to 3294 and 3125 cm^−1^ (O–H/N–H stretch) indicates the presence of hydrogen bonding and covalent interactions between N-CDs and chitosan. The C=O band at 1706 cm^−1^ remained intact, signifying the presence of the carboxyl group, while the amide bond was observed at 1642 cm^−1^. Furthermore, a pronounced peak emerged at 1183 cm^−1^, which is ascribed to C–N, indicating the establishment of an amide bond between the NH_2_ group of chitosan and the COOH group of N-CDs. All these findings confirm that the successful functionalization of chitosan on N-CDs.

**Fig. 3:**
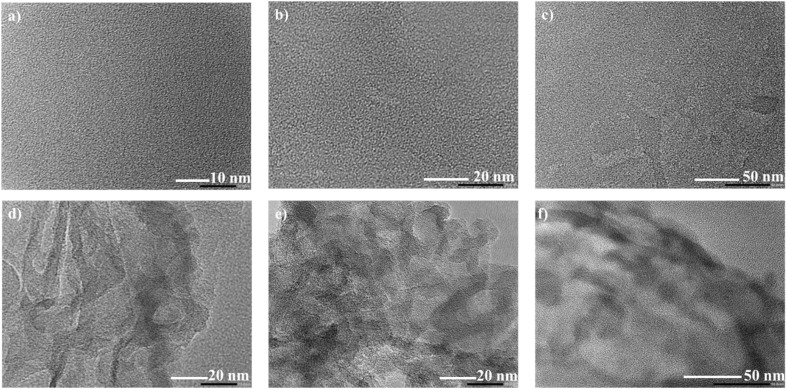
HRTEM images: (a–c) N-CDs with spherical morphology and size of 1–2 nm, and (d–f) N-CDs@C with N-CDs embedded in the chitosan matrix.

The size and morphology of N-CDs and N-CDs@C were examined by HR-TEM. As shown in Fig. 3a–c, N-CDs are spherical in shape with a size between 1–2 nm. The HRTEM images of N-CDs@C showed the fine distribution of N-CDs within the chitosan without any aggregation (Fig. 3d–f). All these results support the successful formation and distribution of N-CDs in N-CDs@C.

**Fig. 4:**
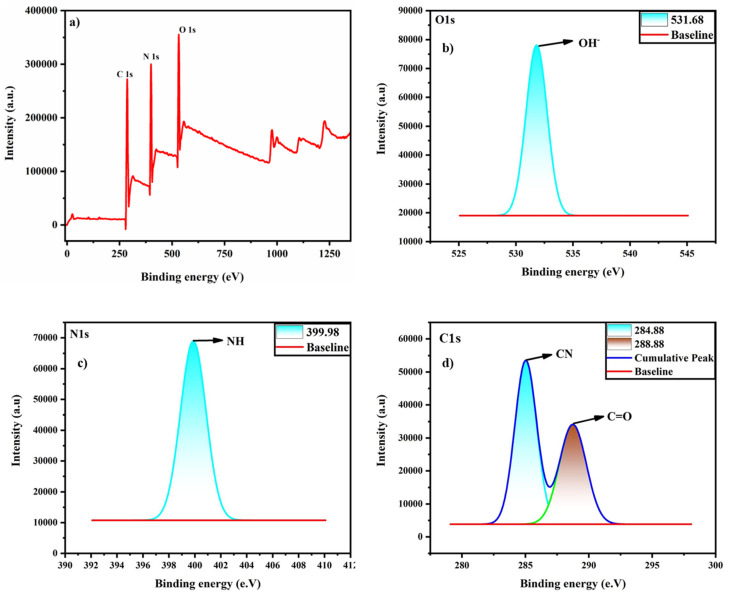
XPS survey spectra of N-CDs@C: (a) full survey spectrum; (b) O1s; (c) N1s; and (d) C1s.

The surface chemical composition and elemental analysis were analysed through XPS and EDS. The full XPS survey spectra show the presence of oxygen (O), nitrogen (N) and carbon (C) (Fig. 4a). In the high-resolution O1s spectra, the one peak at 531.68 eV represents the OH group (Fig. 4b). The N1s spectra show a peak at 399.9 eV, representing the presence of the N–H group (Fig. 4c). In the C1s spectra, two bands at 284.88 and 288.88 eV correspond to C–N and C=O, respectively (Fig. 4d). Further, elemental analysis was confirmed by EDS. C, N, and O are present in the N-CDs with an atomic % of 77.99, 14.99, and 5.73, respectively (Fig. S1a). On functionalization with chitosan, there is the presence of all the elements with increasing atomic % of N, *i.e.*, 19.70% which suggests successful incorporation and a strong interaction between chitosan and N-CDs within the N-CDs@C (Fig. S1b). The multiple amino groups in chitosan contribute to the increase in the nitrogen content in the N-CDs@C. This implies that the nitrogen-containing functional groups of chitosan and the surface sites of the N-CDs have strong chemical or physical interactions, such as covalent or hydrogen bonding.

**Fig. 5:**
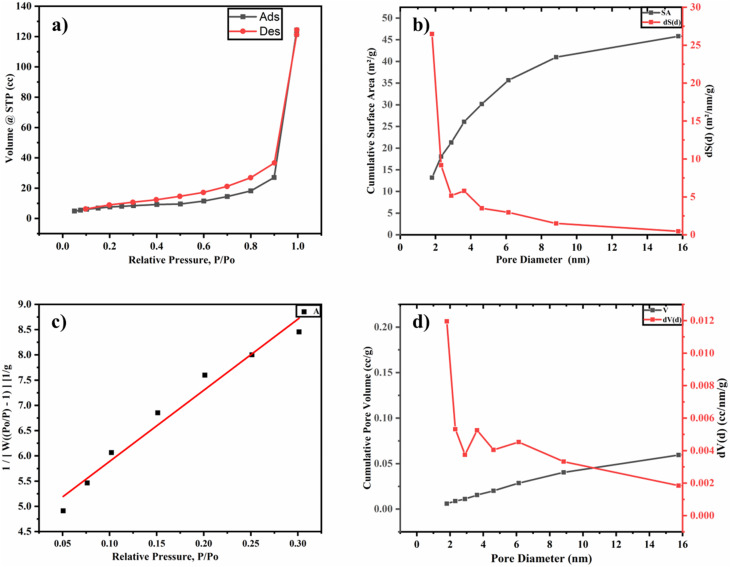
N_2_ adsorption desorption analysis of N-CDs@C: (a) physio–sorption isotherm curve of Type IV; (b and d) BJH pore size distribution curve (1.806 nm); (c) multipoint BET curve (surface area = 48.45 m^2^ g^−1^).

The N_2_ adsorption–desorption analysis was performed to assess the textural properties of N-CDs@C (surface area, pore size, and pore volume). Fig. 5a illustrates that the physisorption isotherm is of Type IV isotherm, showing the mesoporous nature of N-CDs@C. The specific surface area was calculated using a multipoint BET linear plot, employing the slope and intercept derived from the linearized BET equation (*R*^2^ = 0.99), resulting in a value of 48.45 m^2^ g^−1^ (Fig. 5c). The BJH pore volume and pore diameter were found to be 0.356 cc g^−1^ and 1.814 nm, respectively (Fig. 5b and d), while the total pore volume and average pore size were 0.199 cc g^−1^ and 2.83 nm, respectively. This illustrates the high surface area and mesoporous structure of N-CDs@C, which should improve mass transport and offer a large number of active sites, leading to improved F^−^ sensing performance.

### 3.3. Detection of F^−^ by N-CDs@C

**Fig. 6:**
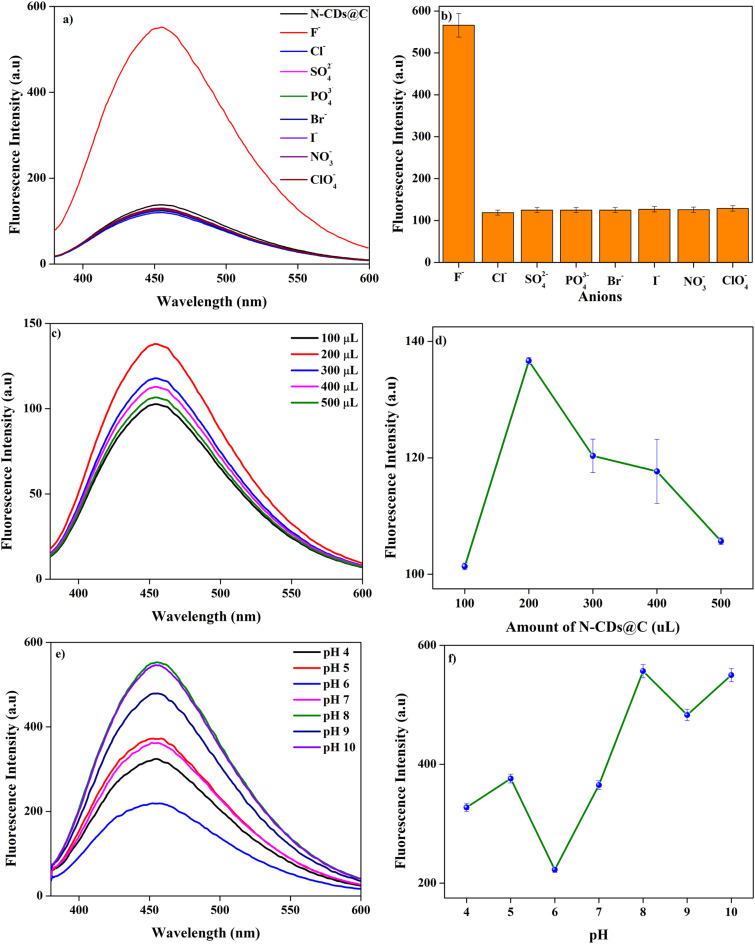
(a and b) Fluorescence emission spectra of N-CDs@C in the presence of different anions; (c and d) Effect of N-CDs@C amount (100–500 μL) on the fluorescence intensity of N-CDs@C; and (e and f) effect of pH (4–10) on the quenching efficiency of N-CDs@C by F^−^.

The sensing efficacy of N-CDs@C was assessed for the fluorescence detection of various anions, including F^−^, Cl^−^, SO_4_^2−^, PO_4_^2−^, Br^−^, I^−^, NO_3_^−^, and ClO_4_^−^ at a concentration of 0.5 μM with an emission wavelength of 455 nm. As illustrated in Fig. 6a and b, among all the anions, only F^−^ showed a significant fluorescence enhancement, indicating the high selectivity of N-CDs@C towards F^−^. The intensity enhancement of N-CDs@C in the presence of F^−^ can be explained by the interaction between F^−^ and functional moieties present on the surface of N-CDs@C. As F^−^ is the most electronegative ion and the smallest ion capable of forming strong hydrogen bonding with the functional groups (–OH, –C=O, –NH_2_). This interaction might lead to surface passivation or defect sites in CDs, which minimizes non-radiative energy loss and enhances radiative transitions, leading to an increase in fluorescence intensity. In addition, the binding of F^−^ can stabilize the excited state CDs and can inhibit the photoinduced electron transfer (PET), thereby facilitating fluorescence enhancement. Comparative sensing studies were also conducted to evaluate the response of N-CDs and N-CDs@C to F^−^ detection. N-CDs@C performed better than N-CDs in terms of enhancement of F^−^, as seen in Fig. S2. The increased amount of active binding sites and surface functional groups due to chitosan functionalization leads to better binding and more effective interaction, which results in greater sensitivity and selectivity towards F^−^ ion.

To enhance the optimization of the sensor, both the amount of N-CDs@C and the pH of the solution have been thoroughly studied. The optimization of various amounts of N-CDs@C, ranging from 100 to 500 μL (5 mg in 5 mL of methanol) has been performed. The maximum fluorescence intensity was determined at 200 μL, which was subsequently employed for further experiments (Fig. 6c and d).

The pH of the solution can significantly influence the interaction of N-CDs@C with F^−^, which in turn can affect the sensing performance of N-CDs@C. For this, the pH range (4–10) was studied, and it was observed that maximum enhancement was observed at pH 8 (Fig. 6e and f). At lower pH (4–6), the –NH_2_, –COOH, and –OH groups undergo protonation, which favours electrostatic interactions with F^−^ ions. However, the fluorescence response is still weak due to the lack of H-bonding and the dominance of the non-radiative process due to the protonation of surface functional groups. Moreover, the F^−^ can be converted to HF in acidic conditions, leading to less availability of F-ions for effective interaction. On the other hand, at weak basic conditions (pH 8), these functional groups get deprotonated to form strong and directional hydrogen bonding with F^−,^ resulting in maximal fluorescence enhancement.

**Fig. 7:**
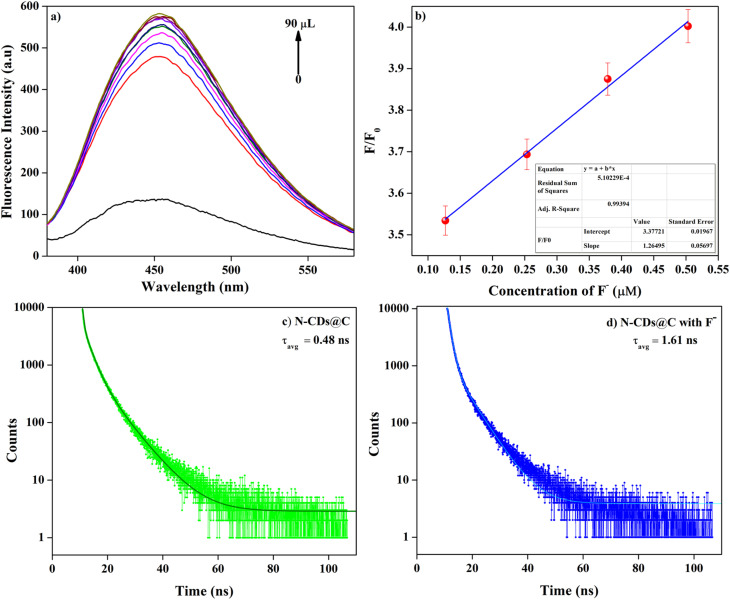
(a) Fluorescence emission spectra of N-CDs@C by gradually adding F^−^ solution (10–90 μL); (b) standard calibration curve at different concentration of F^−^ (0.12–0.50 μM with *R*^2^ value of 0.992); and (c and d) fluorescence lifetime spectra of N-CDs@C in the absence and presence of F^−^.

To assess the sensitivity of N-CDs@C towards F^−^, titration experiments were conducted by gradually introducing 10 μL of F^−^ into the solution of N-CDs@C. As shown in Fig. 7a, there is an increase in enhancement in fluorescent intensity with an increase in the concentration of F^−^. As the F^−^concentration increased, the fluorescence intensity reached a saturation point beyond which no enhancement was observed. This suggests that at higher concentrations, all the interaction sites have been occupied, resulting in a steady signal. A calibration curve has been constructed based on the titration experiment using the normalized fluorescence intensity ratio (*F*/*F*_0_) to minimize variability from experimental conditions and instrumental fluctuations. The calibration curve shows an *R*^2^ value of 0.992 in the concentration range of 0.12–0.50 μM, validating the N-CDs@C quantitative sensing capability by demonstrating that the fluorescence intensity changes proportionately to F^−^ concentration (Fig. 7b). The limit of detection (LOD) was calculated as 0.01 μM based on the 3*σ*/*S* (*σ* = standard deviation of the blank sample and *S* = slope of the calibration curve). This LOD is much lower than the WHO permissible limit value of F^−^ in drinking water (1.5 mg L^−1^ or 79 μM).^37^ This high sensitivity of N-CDs@C indicates that it may detect F^−^ at trace levels that are far below regulatory limits, making it ideal for water monitoring in the real world. This result suggests that the N-CDs@C sensor could detect F^−^ contamination early and respond quickly to guarantee safe drinking water standards.

Moreover, the fluorescence lifetime (*τ*) of N-CDs@C with and without F^−^ was measured using time-resolved photoluminescence spectroscopy. The emission decay profile of N-CDs@C was fitted with a three-component exponential decay curve, while the emission decay profile of N-CDs@C with F^−^ ion was fitted with a two-component exponential decay curve (Fig. 7c and d). The lifetime values for N-CDs@C without and with F^−^ were determined to be 0.48 and 1.61 ns, respectively, indicating an evident change in the dynamics of the excited states upon F^−^ binding.

The sensing efficacy of N-CDs@C towards F^−^ was tested in the presence of different anions (Cl^−^, SO_4_^2−^, PO_4_^2−^, Br^−^, I^−^, NO_3_^−^, and ClO_4_^−^) at a 1 : 1 concentration ratio. As shown in Fig. S3, even in the presence of interferent anions, there is a negligible change in the fluorescence response of N-CDs@C towards F^−^ ion. This demonstrates that the N-CDs@C exhibits exceptional selectivity and specificity for F^−^ even in the presence of potential interfering anions, confirming its robustness and reliability for fluoride sensing applications.

### 3.4. Proposed binding mechanism

**Fig. 8:**
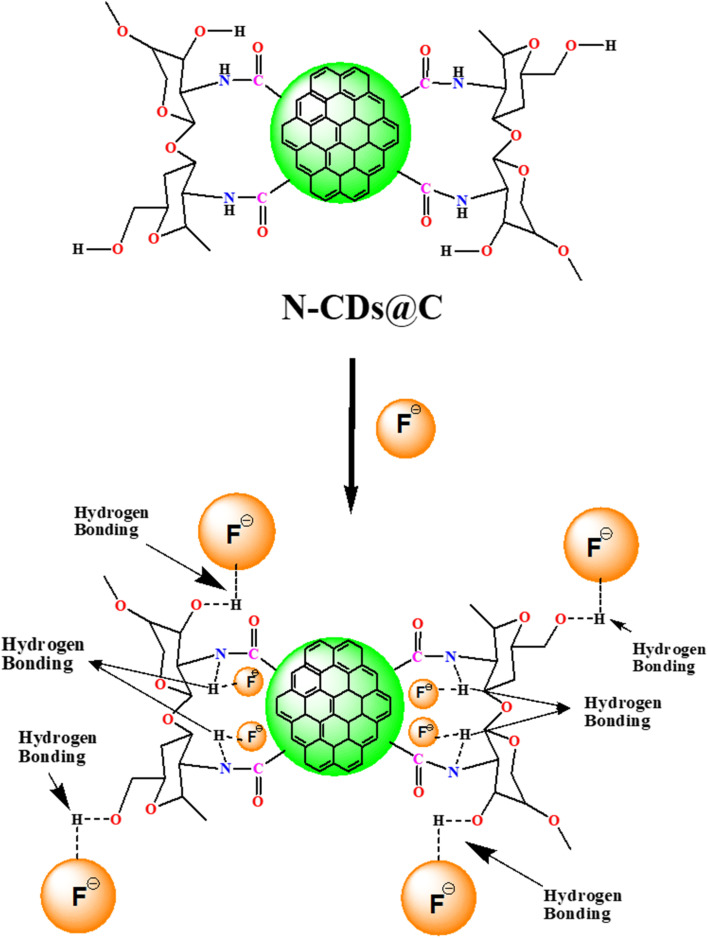
Proposed binding mechanism between N-CDs@C and F^−^.

**Fig. 9:**
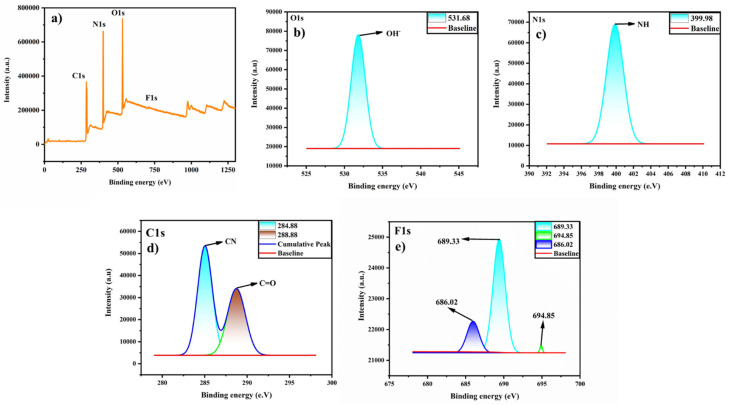
XPS spectra of N-CDs@C after the fluoride binding.

The binding interaction between the N-CDs@C and F^−^ is predominantly hydrogen bonding between the –NH, –OH groups of N-CDs@C and F^−^, leading to the fluorescent intensity enhancement of N-CDs@C in the presence of F^−^ (Fig. 8). This hydrogen bonding stabilizes the surface states and restricts the non-radiative relaxation pathways, resulting in a significant enhancement in the fluorescence intensity of N-CDs@C in the presence of fluoride. The binding interaction between N-CDs@C and F^−^ was further corroborated by FTIR and XPS analysis. The broad O–H and N–H bands around 3125–3294 cm^−1^ shifted to a lower wavenumber (3109–3282 cm^−1^) after the interaction with F^−^. This shift provides a strong indication of H-bonding between –OH and –NH groups of N-CDs@C to form O–H……F and N–H…F, which weakens the bond strength. Additionally, minor changes were observed in the C–H stretch (2795 cm^−1^), C=O (amide/carboxyl) stretching (1640 cm^−1^), and C–N stretching bands (1640 cm^−1^) which indicate alterations in the electronic environment of these functional groups due to F^−^ coordination (Fig. S4). Further XPS analysis confirmed the interaction between the N-CDs@C and F^−^. After binding with F^−^, new peaks appeared at 681.1–689.3 eV, confirming the interaction of F^−^ with N-CDs@C, corresponding to hydrogen-bonded F^−^ ions likely to exist between –COOH and –NH_2_ groups of N-CDs@C and F^−^ (Fig. 9).

### 3.5. Real sample analysis and performance comparison

For the practical applicability of N-CDs@C, tap water and toothpaste samples were taken for the detection of F^−^. Both tap water and toothpaste were detected prior to spiking. F^−^ was detected in the toothpaste sample at a concentration of 0.039 μM. F^−^ was not detected in the water samples, as they might be present below the detection limit of the present method. Tap water was spiked at a known concentration (0.12, 0.37, and 0.62 μM), and fluorescence was recorded under optimized conditions. As shown in Table 1, the recovery values ranged between 98.67–99.27% with RSD between 0.66–1.16%. This demonstrates the potential of N-CDs@C for real-time application of F^−^.

**Table 1:** Real sample analysis of F^−^ in toothpaste and tap water by N-CDs@C

Order	Matrix	Amount added (μM)	Found (μM)	Recovery (%)	RSD (%)
1	Toothpaste	0	0.03	—	2.38
2	Tap water	0.12	0.11	99.16	1.16
		0.37	0.36	98.67	0.96
		0.62	0.61	99.27	0.66

A comparative analysis of different sorbents, including zirconium porphyritic luminescent metal–organic framework (MOF), MOF-UiO-66(NH), fluorescent carbon nanodots, Fe_3_O_4_@SiO_2_@carbon quantum dot, carbon dots/gold nanoparticles, Tb(iii)–CuNCs, blue/yellow emissive carbon dots coupled with cerium, and carbon dots/Fe^3+^ composites, has been conducted for the detection of F^−^. It was observed that most of the materials, including metal organic frameworks and hybrid nanocomposites, demonstrate a detection limit ranging from 0.06–110 μM. In comparison, N-CDs@C showed a better detection limit of 0.01 μM. Conversely, zirconium MOF (0.048–0.065 μM) and Tb(iii)–CuNCs (0.01 μM) exhibited comparable detection limit; however, this necessitates a complex synthesis process. In addition, N-CDs@C achieved good recoveries with RSD <2% in toothpaste and tap water. These results confirm that the N-CDs@C composite is an exceptionally sensitive, selective, and environmentally friendly fluoride sensor for real-world applications (Table 2).

**Table 2:** Comparative analysis of the different sorbents reported for the detection of fluoride anion

Order	Matrix	Sorbent	Linear range (μM)	Detection limit (μM)	Recovery (%)	RSD (%)	Ref.
1	Fresh lake water and tap water	Zirconium porphyritic luminescent metal–organic framework (PCN-222)	1–20	0.048–0.065	88.0–105.6	<5	38
2	Tap water	Metal–organic framework UiO-66(NH)	2–150	0.57	104.10–118.81	<6	1
3	River water and tooth paste	Fluorescent carbon nanodots	0–0.0267	110	97.0–105.9 and 96.1–107.7	<1	39
4	Tap water	Fe_3_O_4_@SiO_2_@carbon quantum dot based nanostructure	1–20	0.06	96	—	40
5	Tap water and river water	Carbon dots/gold nanoparticles hybrid material using 3-mercapto-l,2-propanediol (MP)	9–117	1.5	100.8 and 47.83	<1	41
6	Toothpaste	Tb(iii)–CuNCs (aggregation-induced emission copper nanoclusters)	0.01–0.3	0.01	—	—	22
7	Tap water and milk	Blue/yellow emissive carbon dots coupled with cerium	2–60	0.39	95.1–107.8 and 92.6–109.5	<8	42
8	Aqueous solution	Carbon dots (from wheat straw)	0–0.0015	49	—	—	43
9	Tap water	CQDs/Fe^3+^ from coal washery rejects	0–76.49	1.14	96–100	—	44
10	Toothpaste and tap water	Nitrogen-doped carbon dots functionalized chitosan	0.12–0.50	0.01	98.67–99.27	<2	This work

## 4. Conclusions

In conclusion, nitrogen-doped CDs were successfully prepared by a solid pyrolytic method, and functionalized with chitosan to develop N-CDs@C as a highly sensitive fluorescent probe for selective detection of fluoride ions in aqueous media. The N-CDs@C demonstrate excellent selectivity and anti-interference performance and a distinct fluorescent “turn on” response at 455 nm upon fluoride binding, likely driven by hydrogen bonding or electrostatic interaction. The N-CDs@C achieved a detection limit as low as 0.01 μM within a linear range of 0.12–0.50 μM, which is much better than previously reported hybrid nanocomposites. The practical utility of this sensor was demonstrated through successful application in real samples, including toothpaste and tap water.

## Conflicts of interest

There are no conflicts to declare.

## Supplementary Material

RA-015-D5RA04745E-s001

## Data Availability

All experimental data supporting the findings of this study, including spectral data and characterization results, are provided within the manuscript and the SI. No datasets, software, or code were generated or analyzed beyond the experimental procedures described. List of chemicals and their make required for the synthesis (Table S1); details of the instruments applied for the characterization and analysis of N-CDs and N-CDs@C (Table S2); EDS spectra of the synthesized materials: (a) N-CDs and (b) N-CDs@C (Fig. S1); comparative analysis of the sensing performance of N-CDs and N-CDs@C towards F^−^ ion (Fig. S2); fluorescence emission intensity of N-CDs@C in the presence of F^−^ and other interfering anions at a 1 : 1 ratio concentration (Fig. S3); and comparison of FTIR spectra between N-CDs@C before and after binding with F^−^ (Fig. S4). See DOI: https://doi.org/10.1039/d5ra04745e.
