# Supplementary material for: Chitosan functionalized nitrogen-doped carbon dot nanocomposites: a turn-on sensor for fluoride detection
Source: RSC Adv. 2025 Sep 17;15(41):34094–104. doi: 10.1039/d5ra04745e (PMC12442973; doi:10.1039/d5ra04745e)
Supplement: RA-015-D5RA04745E-s001 [file RA-015-D5RA04745E-s001.pdf]

Supplementary Information (SI) for RSC Advances.
This journal is © The Royal Society of Chemistry 2025

Chitosan functionalized nitrogen-doped carbon dots nanocomposites: A turn-on sensor for fluoride detection

Sujal [a], Raj Singh [a], Suman Swami [a*], and Shikha Bhogal [b*]

[a] *Department of Chemistry, Chandigarh University, Gharaun, Mohali, Punjab, India-140413*

[b] *Virginia Tech-TIET Center of Excellence in Emerging Materials, Thapar Institute of Engineering & Technology, Patiala – 147004, India*

*Corresponding author: sumanswami1994@gmail.com (S.S.); shikha.bhogal@thapar.edu (S.B.)

Table S1. List of chemicals and their make required for the synthesis.

Order	Chemicals Name	Make
1.	Ethylene diaminetetracetic acid	Sigma-Aldrich
2.	Urea	Loba Chemie
3.	Chitosan	Loba Chemie
4.	Tetrabutylammonium fluoride hydrate	Sigma-Aldrich

Table S2. Details of the instruments applied for the characterization and analysis of N-CDs and N-CDs@C

Order	Technique	Model
1.	Fourier transform infrared (FTIR) spectra	PerkinElmer Spectrum Two FTIR Spectrometer
2.	X-ray photoelectron spectroscopy	Thermofisher scientific Nexus base

	(XPS)	XPS
3.	Brunauer-Emmett-Teller (BET) surface area analyzer	Autosorb iQ Station 2 system, Quntachrome Instruments, USA
4.	High- resolution transmission electron microscopy (HRTEM)	JEOL JEM 2100 Plus, JEOL, Japan
5.	Energy dispersive X-ray spectra (EDS)	JEM 2100 plus, JEOL, Japan
6.	Fluorescence Spectra	Shimadzu Fluorescence RF-6000 equipped with a quartz cell
7.`	Life time measuring system	Delta Flex TCSPC system (Horiba)

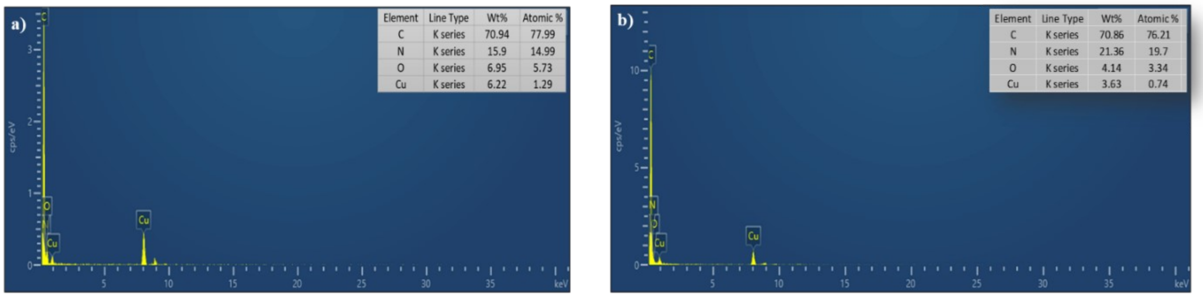

Figure S1. EDS spectra of the synthesized materials: a) N-CDs and b) N-CDs@C

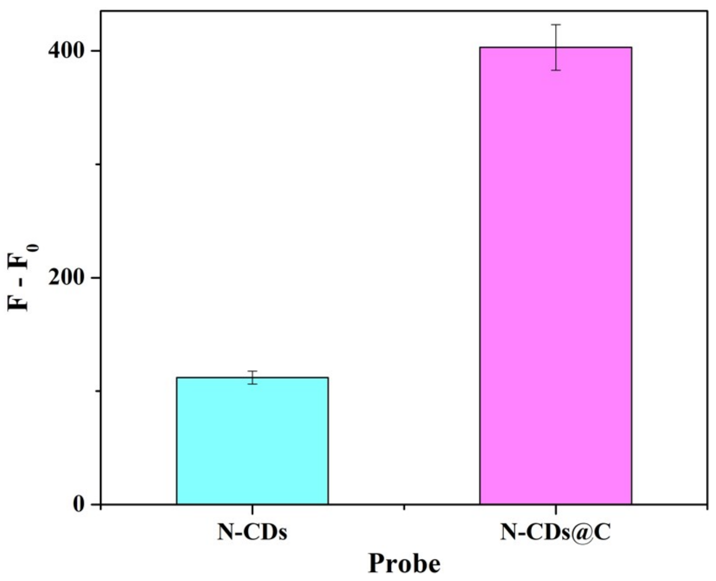

Figure S2. Comparative analysis of the sensing performance of N-CDs and N-CDs@C towards F^- ion

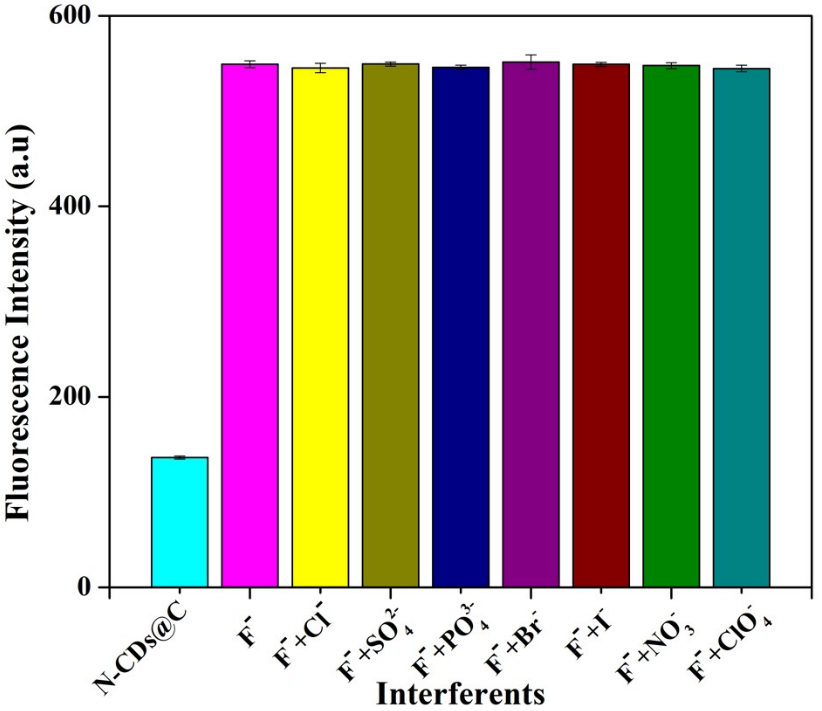

Figure S3. Fluorescence emission intensity of N-CDs@C in the presence of F^- and other interfering anions at a 1:1 ratio concentration

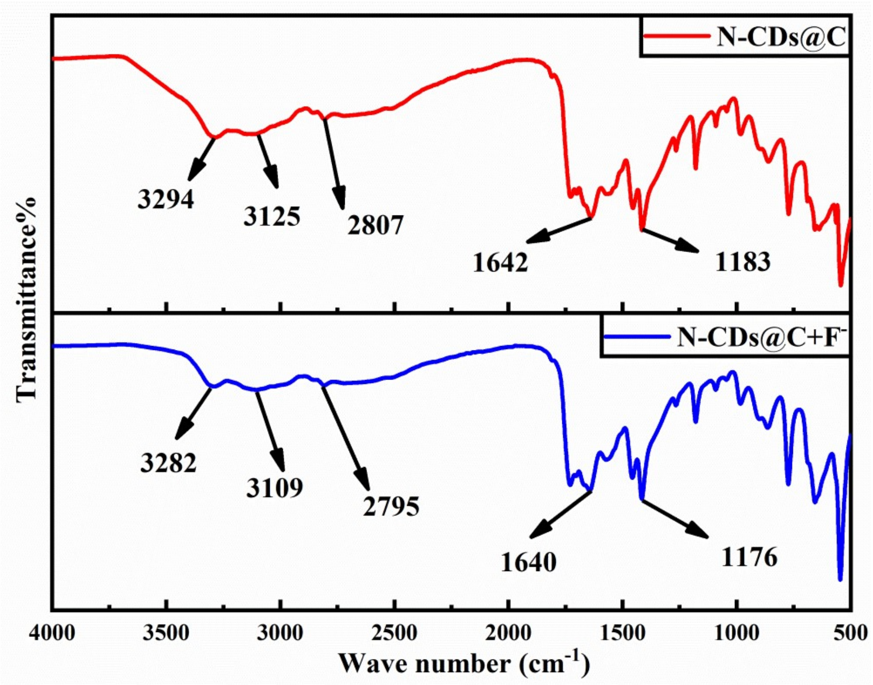

Figure S4. Comparison of FTIR spectra between N-CDs@C before and after binding with F^-
